# Towards Detecting Associations of Canine Astrovirus and Caliciviruses with Health and Living Characteristics of Dogs in Greece

**DOI:** 10.3390/pathogens14010092

**Published:** 2025-01-18

**Authors:** Efthymia Stamelou, Konstantinos Papageorgiou, Dimitrios Papadopoulos, Georgios Delis, Dimitrios Chatzopoulos, Zoi Athanasakopoulou, Efstratios Moschidis, Evanthia Petridou, Spyridon K. Kritas

**Affiliations:** 1 Laboratory of Microbiology and Infectious Diseases, School of Veterinary Medicine, Faculty of Health Sciences, Aristotle University of Thessaloniki, 54124 Thessaloniki, Greece; efistamel@hotmail.gr (E.S.); dpapvet@hotmail.com (D.P.); epetri@vet.auth.gr (E.P.); skritas@vet.auth.gr (S.K.K.); 2 Laboratory of Pharmacology, School of Veterinary Medicine, Faculty of Health Sciences, Aristotle University of Thessaloniki, 54124 Thessaloniki, Greece; delis@vet.auth.gr; 3 Faculty of Public and One Health, University of Thessaly, 43100 Karditsa, Greece; dchatzopoulos@uth.gr; 4 Laboratory of Microbiology and Parasitology, Faculty of Veterinary Medicine, University of Thessaly, 43100 Karditsa, Greece; zathanas@uth.gr; 5 Centre for Research and Technology Hellas (CERTH), 57001 Thessaloniki, Greece; smos@uom.edu.gr

**Keywords:** astrovirus, sapovirus, dogs, multiple correspondence analysis, ascending hierarchical classification

## Abstract

Astroviruses and caliciviruses are important causative agents of gastroenteritis in humans worldwide. They have been detected in a variety of animal species, including dogs, but their role in the induction of disease in animals remains uncertain. In a molecular study that was conducted in Greece, including healthy and gastroenteritis-affected dogs of different ages, astrovirus (AstV) and sapovirus (SaV) were detected in 15% and 26% of the examined animals, respectively. A specialized questionnaire was filled out for each of the dogs participating in the study, including information about different characteristics and risk factors that could possibly affect their health status. This information was analyzed with the use of two innovative statistical methods, i.e., a Multiple Correspondence Analysis (MCA) and the Ascending Hierarchical Classification (AHC). Based on their results, it was possible to define various groups of dogs based on their characteristics. AstV seems to occur more often in low-health-status dogs, usually mongrels, living in rural areas, showing vomit, diarrhea, and diet changes. Dogs of this group usually live with other pets in the same household and have frequent contact with stray animals. The presence of SaV does not seem to be associated with any of the examined factors.

## 1. Introduction

Canine infectious gastroenteritis is a worldwide problem, as it is one of the most common reasons why dogs visit a veterinary clinic and for hospitalization. Determining the cause of gastroenteritis can be very challenging for the veterinarians, as there are several agents which could induce it, such as viruses, bacteria, or protozoans [1]. Viruses are common aetiologic agents of canine infectious gastroenteritis, having been detected in 40–60% of cases of gastroenteritis in dogs, as evidenced in several studies [2,3,4]. Astrovirus, norovirus, and sapovirus are important enteric viruses, with a worldwide distribution. They have been reported in many species, including humans, swine, mink, cats, birds, and dogs [5,6,7,8,9,10].

Astroviruses are members of the *Astroviridae* family, which consists of two genera, namely Mamastrovirus and Avastrovirus [11]. They are in general non-enveloped, single-stranded, positive-sense RNA viruses [12]. Members of the genus Mamastrovirus infect various mammals, including humans [13], bovine [14], feline [15], porcine [16], and mink [17], while members of the genus Avastrovirus infect mainly avian species, such as chicken, turkey, and duck [18,19,20]. Astrovirus is estimated to cause approximately 10% of gastroenteritis cases in children worldwide and was first detected in 1975 in the feces of infants [21]. Canine astrovirus (AstV) was detected for the first time in 1980 in the USA in the feces of beagle puppies with diarrhea [22]. Since then, AstV has been detected in a lot of countries worldwide [23,24,25,26,27]. The high genetic diversity of AstV makes diagnosis and future control strategies very challenging [28,29]. AstV has been detected at higher rates in symptomatic dogs with gastroenteritis than in asymptomatic ones, thus indicating a potential role in canine diarrhea [30,31]. However, the association of the virus with clinical disease still remains uncertain.

Norovirus (NoV) and sapovirus (SaV) are non-enveloped, single-stranded, positive-sense RNA viruses and are members of the family *Caliciviridae* [32]. Caliciviruses were detected in dogs in 1985 in the USA for the first time [33]. Canine NoV was first detected in Italy in 2007 in a puppy with signs of gastroenteritis [34]. Since then, canine NoV has been detected in the feces of asymptomatic and symptomatic dogs in a lot of countries around the world [9,35,36,37,38,39]. Regarding the epidemiology of canine SaV, there are very few data available. The virus has been detected in the USA, Japan, and Italy, with prevalence varying from 0.25 to 2.2% [39,40,41].

The presence of these viruses in dogs and their association with various parameters such as the living and hygienic conditions of the animal have not been thoroughly investigated.

In the present study, we examined the association of the current presence of canine AstV and caliciviruses detected in dogs in Greece [9] with parameters that might affect their health status.

## 2. Materials and Methods

This study was approved by the Institutional Review Board of the School of Veterinary Medicine, Faculty of Health Sciences, Aristotle University of Thessaloniki (protocol code 75/19-Jan-2017).

### 2.1. Characteristics of the Study Population

In Greece, 40% of households are estimated to have at least one pet, 14% of which own dogs. It is estimated that there are around 6000 pet dogs and 3 million stray dogs and cats in the country [42,43]. This study took place from January 2017 to May 2018.

### 2.2. Selection of Veterinary Clinics and Sampling

**Figure 1:**
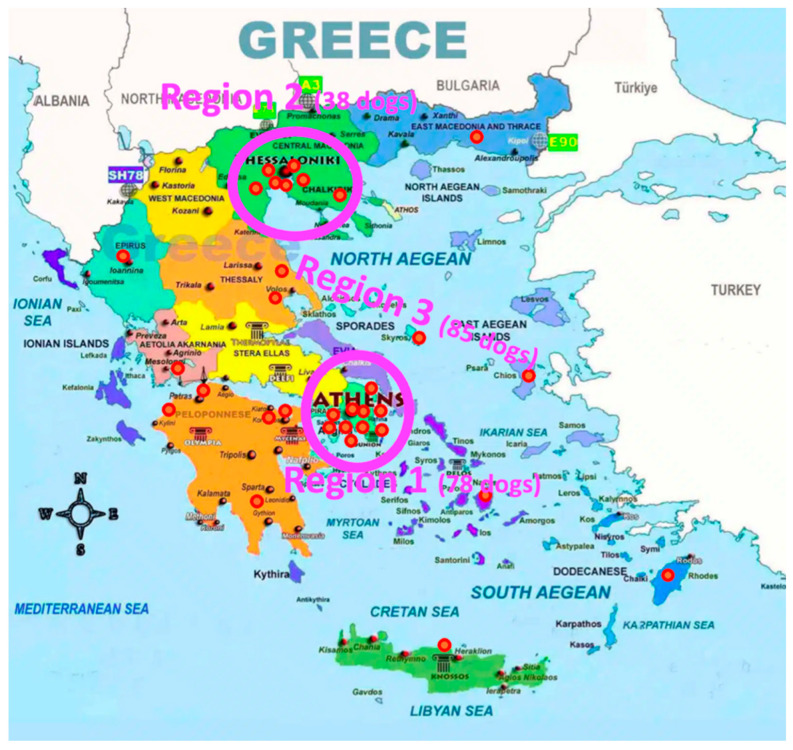
Map of Greece indicating the sampling areas (veterinary clinics) as red dots.

Thirty-three (33) veterinary clinics were randomly selected from various areas around Greece (Figure 1). Veterinary practitioners in these clinics were requested to sample dogs equally from two age groups, e.g., younger or older than 12 months of age, that were either ill (preferably with signs of gastroenteritis) or healthy at a roughly equal ratio. Two hundred and one (201) pet dogs were eventually sampled. Two swab samples were obtained from each dog, one saliva sample and one fecal sample. After the sampling, the swab was inserted in a 1.5 mL Eppendorf tube, which contained 1 mL of the RNAlater (Sigma Aldrich, St. Louis, MO, USA) solution for the stabilization of RNA, and was transferred to the lab in ice in isothermic boxes. When arriving at the lab, each sample was vortexed for 5 min and centrifuged at 12,000× *g* for 10 min. The supernatants were collected, pooled together in pools of 3–5 (which were placed in new sterile 1.5 mL Eppendorf tubes), and stored at −80 °C until further processing.

### 2.3. Collection of Dog Information

A specialized questionnaire was created for the dogs’ owners in order to extract information about different characteristics and parameters that could possibly affect the health status of their dogs. The questionnaire was delivered to the veterinarians, who were filling it in after communication with the dogs’ owners. The obtained information pertained to the following:

- Geographical region (1—Attiki/Athens, 2—Central Makedonia/Thessaloniki, 3—rest of Greece).
- Dog’s age (<12 months, ≥12 months).
- Breed (purebred, mongrel).
- Gender (male, female).
- Body condition [underweight (weighing less than 15% of their ideal body weight), normal, overweight (weighing more than 15% above their ideal body weight)] [44].
- Presence of diarrhea (yes, no).
- Presence of vomit (yes, no).
- Any change in dog’s diet during the last month (yes, no).
- Dog on standard vaccination program (Canine Distemper virus, Canine hepatitis, Canine Parvovirus Disease, Leptospirosis, Parainfluenza, and Rabies) (yes, no).
- Dog on standard ectoparasitic program (fleas, lice, and ticks) (yes, no).
- Dog on standard endoparasitic program (worms, tapeworms, dirofilaria, and Leishmania) (yes, no).
- Any simultaneous symptoms in a human member of the family (yes, no).
- Presence of other animals on the family premises (yes, no).
- Dog’s regular contact with stray dogs or other animal species (yes, no).

### 2.4. Laboratory Testing

Total RNA was extracted from a 200 μL volume pooled sample using the RNA extraction kit “Cador pathogen kit” (Qiagen, Germany). A Spectrophotometer (Eppendorf) was used to evaluate the quality and quantity of the extracted RNA. The extracted RNA was stored at −80 °C until further analysis. The samples were tested both with conventional and SYBR Green real-time RT-PCR for the detection of AstV, NoV, and SaV after the RNA extraction as presented elsewhere [9]. Briefly, three different primer pairs were used for the detection of canine NoV and SaV by conventional RT-PCR. The first was the universal primer pair p289-p290 that targets a conserved region of caliciviruses and detects both NoV and SaV [45]. The second and third primer pairs detect NoV (HΚ NORO-F (5′-RHYATTGACCCCTGGATW-3′) and HK NORO-R (5′-AACGCATTCCCHGCMARKA-3)′ [46] and SaV (DogSap1F (5′-ACACACGATCCAAATTCACCAA-3′) and DogSap1R (5′-TGCCAGACAGACCTCCAATTG-3′) [40], respectively. Canine AstV was detected using conventional PCR (with the use of primers 625F (5′-GTACTATACCRTCTGATTTAATT-3′) and 626R (5′-AGACCAARGTGTCATAGTTCAG-3′) [24]. SYBR Green real-time RT-PCR was performed for canine AstV with the use of primers F2-R2 (F2: 5′-TTCCCTGCTTCTGATCAG-3′ and R2: 5′-CTCACTTAGTGTAGGGAGAG-3′) [47], for canine NoV with the use of primers F: GCTGGATGCGGTTCTCTGAC and R: TCATTAGACGCCATCTTCATTCAC [46], and for canine SaV with the use of the DogSap1F-DogSap1R primer pair [40].

Samples that were positive for AstV or caliciviruses were sequenced and further processed using MEGA-X software (MEGAX _10.0.5_64bit version) after a similarity assessment by the Basic Local Alignment Search Tool (BLAST) [48]. Their results were already published [9].

### 2.5. Statistical Analysis

The nature of the data collected through questionnaires is such that unsupervised learning methods are particularly suitable for discovering trends and patterns among categories of qualitative variables. Also, a common exploratory stage is the clustering of objects and the characterization of their profiles. Suitable methods for these tasks are Multiple Correspondence Analysis and Ascending Hierarchical Classification. [49,50,51].

A Multiple Correspondence Analysis (MCA) interprets the direction of the variation in a phenomenon and trends that develop between the categories of the variables, using the concept of inertia:

$$I=\sum_{i}m_{i}d_{i}^{2}$$

where

- I is the total inertia.
- $m_{i}$ is the mass (the relative frequency) of observation i.
- $d_{i}^{2}$ is the squared distance from the center of gravity.

In the MCA, as is customary, we present the first two principal dimensions, which correspond to the first two factorial axes derived from the method. This is further supported by the corresponding scree plot, which illustrates the percentage of inertia explained by each factorial axis.

The Ascending Hierarchical Classification (AHC) is a powerful unsupervised data analysis technique that clusters objects based on their similarity by producing a hierarchy of nested clusterings rather than a single flat partition of the data compared to partitional clustering methods, such as K-means [52]. The agglomeration criterion used was Ward’s method. The metric distances were calculated using the Euclidean metric for the points and their coordinates within the new coordinate system of the factorial axes, as derived from the MCA.

The MCA method was implemented in scripts performed with R [53], an open-source programming language and environment for statistical computing and graphics. Specifically, the main library used to implement the methods and obtain the results was FactoMineR [54]. The AHC was written in Python [55], and the main library used scikit-learn [56]. These methods were launched by their default configuration and using a personal computer.

As the variable Nov was not included in the analysis, the exclusion of related interpretative coordinates, interpretative axes, and interpretative planes was automatically managed using the approach outlined in [51] and the recently introduced (2022) concept. This ensures a more straightforward and accurate visualization and interpretation of the results compared to the classical MCA approach

## 3. Results

The characteristics of the sampled dogs are summarized in Table 1. All age and gender groups were almost equally represented. Almost half of the dogs were fully dewormed for ectoparasites, and half of the dogs were living with other animals in the household. Most of the dogs were purebred (57.2%), in normal body condition (69.7%), fully vaccinated (80.6%), fully dewormed for endoparasites (72.6%), and did not have contact with stray animals (68.7%).

Of the sampled animals, 38.3% showed diarrhea and 22.4% showed vomit. A change in diet was reported in 17.4% of the dogs. Finally, in 3% of the dog households, signs of illness were present to members (humans) of the family during sampling.

### 3.1. MCA

To interpret the factorial axes, we used a new method for visualizing the results of the MCA [51], which is sufficient to show the significance of points on a factorial axis. According to the authors, the concepts of an interpretive coordinate, interpretive axis, and interpretive plane are developed. Reading the value of the interpretive coordinate shows both the direction on the corresponding factorial axis (from the sign) and the importance of the point on the corresponding factorial diagram.

**Figure 2:**
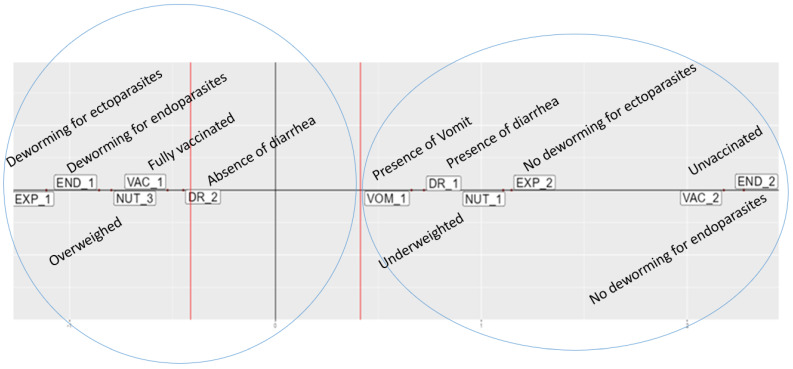
First interpretive axis F1 (x) showing F1A group (left side) and F1B group (right side).

**Figure 3:**
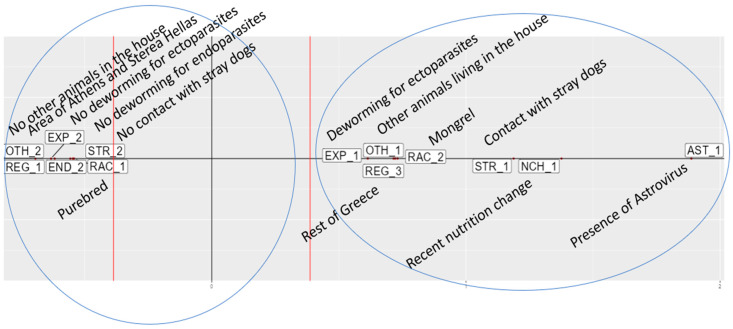
Second interpretive axis F2 (y) showing F2A group (left side) and F2B group (right side).

The projection of the viruses with respect to the other dominant variables (e.g., those having a contribution greater than the average contribution toward the creation of each axis) is depicted in a two-dimensional factorial plane, defined by the factorial axes F1-x and F2-y, presented in Figure 2 and Figure 3, respectively. Two tendencies (groups) of dogs, with roughly contrasting characteristics, were observed on each axis. It should be noted that in none of these groups was the presence of SaV involved as an important feature.

In the F1 (interpretive x) axis (Figure 2), a group of dogs (F1A) that were generally of a good health status was observed. These dogs were properly dewormed for endo- and ectoparasites, fully vaccinated, overweight, and without any signs of diarrhea. A second group of dogs of a lower health status (F1B) was formed, including underweight animals that were not properly vaccinated or dewormed with a history of diarrhea and vomit.

In the F2 (interpretive y) axis (Figure 3), two groups of dogs and characteristics were identified: A) a group of dogs (F2A) with the following characteristics: living in the area of Athens, purebred, no contact with stray dogs or other animals in the house, and without deworming treatment. B) a group of dogs positive for AstV (F2B), mainly mongrels, living with other animals in the house, in frequent contact with stray dogs and with recent changes in their nutrition, dewormed for ectoparasites, and localized in more rural areas of Greece.

### 3.2. AHC

**Figure 4:**
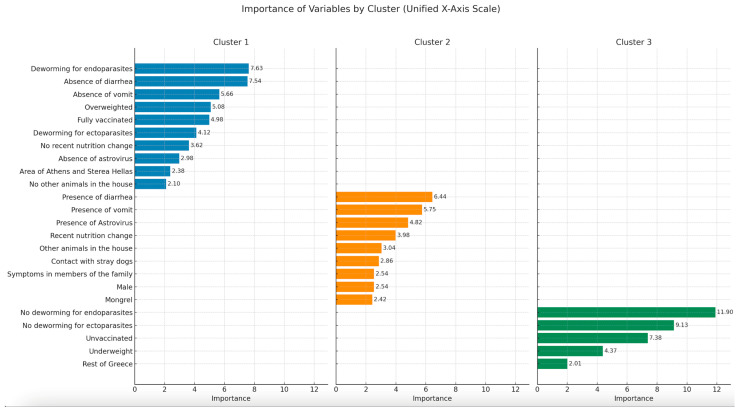
Summarizing importance of variables by cluster (unified X-axis scale).

In the AHC, three different groups (clusters) of dogs (based on their homogeneity) were formed (Figure 4). It should be noted that in none of these groups was the presence of SaV involved.

The first cluster of dogs, Cluster 1 (Figure 4), included dogs negative for AstV, fully dewormed and vaccinated, living in households without other animals, and in the urban area of Athens. These dogs were overweight and had no clinical signs of vomit or diarrhea, nor any recent change in their nutrition.

The second cluster, Cluster 2 (Figure 4), included male mongrel dogs positive for Astrovirus, living in households with other animals, and having contact with stray dogs. These dogs showed signs of diarrhea and vomit and had recent changes in their diet. Humans of the family with which these dogs lived presented symptoms.

The third cluster, Cluster 3 (Figure 4), included not or partially dewormed or vaccinated underweight dogs living in more rural areas of Greece.

## 4. Discussion

Several members of the *Astroviridae* and *Caliciviridae* families are involved in human diarrheal disease [57]. Members of these families, but different than those observed in humans, may also affect dogs [9]. As the presence of AstVs and caliciviruses in dogs, either as causative agents or as contributors of disease, has not been well documented so far, it was considered interesting to investigate under which conditions such viruses can be detected in this animal species.

When investigating risk factors in a new unknown field containing several categorical variables, it may be difficult to detect interactions or/and correlations of each parameter with the other, as there might be unknown inherent and less visually expected multi-interactions. The use of conventional statistics that show the statistical strength amongst the relation of two factors may not be the ideal solution, at least at the beginning of the research. For these reasons, we investigated the possibility of interpreting our results with the use of two different techniques, both employed in the interpretation of big groups of datasets in social sciences, biology, and computer sciences [58,59,60]. These methods interpret results based on different principles, without the need for the preliminary determination of any dependent values: the MCA by calculating the distances between the variables (inertia) after taking into account their relative weight in the entire database, and the AHC by examining the similarity of variables through producing a hierarchy of nested clusterings [52]. Both methods point out reasonably defined risk factor groups based on their characteristics and may help uncover hidden correlations between variables that would otherwise remain undetected. In addition, they provide valuable insights into behavior or trends.

Initially, the absence or presence of gastrointestinal signs may determine good- or low-health-status dogs, respectively. Good-health-status dogs show characteristics interpreted both by the MCA (group F1A) and AHC (Cluster 1). These dogs are fully dewormed and vaccinated, overweight, without any recent diet change, living in the urban area of Athens, and in households without other pets. On the other hand, low-health-status dogs may show characteristics interpreted by the MCA (group F1B) and by the AHC (Cluster 2). These dogs are underweight animals and not properly vaccinated or dewormed. They are usually male mongrel dogs, positive for AstV, living in households with other pets, having contact with stray dogs, and with recent changes in their diet, while humans in the same household may show symptoms.

Thus, it seems that AstV-affected dogs (grouped by both MCA-F2B and AHC-Cluster 2) can also share many characteristics found in low-health-status grouped dogs (e.g., with signs of diarrhea or/and vomit), while the characteristics of AstV-negative dogs appear to be shared with dogs of a good health status (e.g., without signs of diarrhea or/and vomit).

The interpretation and explanation of many of these findings, as well as of their grouping, could be attributed to the dog-living characteristics of Greece. In intensely urbanized areas, dogs live confined to the owner’s premises, usually an apartment, with a couple of daily hourly walks that permit limited exercise (more overweight animals) and with only limited contact with other pets or a few existing stray dogs that may harbor microbial agents. On the other hand, in less urbanized areas (e.g., region 2 and particularly region 3), dogs live either in apartments or in houses with yards together with other pets as space is not an issue, many times without the need for restricted daily walks, and have chances for more contact with frequently found stray dogs. Indeed, dogs in rural areas are less restricted and less supervised by their owners (this may also imply less meticulous deworming and vaccination) when compared to dogs living in flats and apartments in urban areas. Thus, rural dogs may be freer to approach stray dogs (particularly male dogs, which are prone to be more aggressive and therefore come in closer contact through fighting and smelling other dogs) and even breed with them, and this is why mongrels are more common in rural than in urban areas. This may also explain the higher incidence of AstV in male mongrel animals. Other researchers have also observed that AstV is common in crossbreed dogs [24,31]. In addition, stray dogs are usually unvaccinated and of lower health status, possibly harboring various microorganisms at higher doses and serving as a source to other dogs coming into contact with these microorganisms.

AstV presence is also observed to be associated with diet changes. As the chronical sequence of such changes (before or after the sampling) was not noted, it was not clear whether these changes triggered the infection itself or were caused by it. Diet changes could have caused stress to a dog, resulting in the decline in its immune system and thus making the dog more vulnerable to microorganisms, including astroviruses. On the other hand, the loss of body weight, vomit, and diarrhea are usual clinical signs of enteric viruses, and diet changes are necessary to recover from such illness.

The enteric virome of dogs has not been studied in general [61]. A metagenomic analysis that was performed in 2017 in samples of healthy and diarrheic dogs showed that *Astroviridae* and *Caliciviridae* are not part of the enteric virome of dogs, as they were detected only in dogs with acute diarrhea [61]. Interestingly, *Astroviridae* was the most frequently detected virus family [61]. In our study, although SaV was detected in a higher frequency compared to AstV (26% versus 15%), it seems that SaV is not associated with the examined risk factors as it was not included in any group or cluster. It could be that associations between SaV and examined risk factors were not as powerful as those of AstV with these factors.

Finally, it was interesting to observe clinical signs in a few humans living in the households of low-health-status dogs. Although none of these human cases were associated with AstV presence, other microorganisms (not examined at present), possibly found in low-health-status dogs, could have been responsible for such symptoms in humans.

Cross-species transmission of both avian AstVs and Mamastrovirus has been described, attributed probably to recombination events [12,19,62,63,64,65,66,67]. Humans have been brought into close contact with both avian and mammalian AstVs due to domestication [68]. Multiple recombination events between porcine AstVs and human AstVs have been reported [66]. There is a debate about the directionality of this transmission, indicating that transmission from humans to livestock could be occurring in addition to traditional zoonosis [68]. Recombination events have also occurred between human and feline AstVs [69], but the occurrence of such events has not yet been described between humans and dogs. Nevertheless, although no connection was traced between the investigated viruses in the current study, clinical signs in dogs, and specifically those living under low-health-status conditions, may always be considered when a disease arises in humans in the same household.

## 5. Conclusions

By using the MCA and AHC, it was possible to define various groups of dogs based on their characteristics. AstV seems to occur more often in low-health-status dogs, usually mongrels, living in rural areas, showing vomit, diarrhea, and diet changes. Dogs of this group usually live with other pets in the same household and have frequent contact with stray animals. On the other hand, the presence of SaV does not seem to be associated with any of the examined factors.

## Figures and Tables

**Table 1:** Characteristics of the dogs (%) participating in the study.

Variables	Code	Values	Dogs (%)					
			1		2		3	
Geographical region	REG	1 (Central Greece/Athens), 2 (Central Macedonia/Thessaloniki), 3 (rest of Greece	78	(38.8)	38	(18.9)	85	(42.3)
Age	AG	1 (<12M), 2 (≥12M)	92	(45.8)	109	(54.2)		
Gender	GEN	1 (male), 2 (female)	101	(50.3)	100	(49.7)		
Breed	RAC	1 (purebred), 2 (mongrel)	115	(57.2)	86	(42.8)		
Body condition	NUT	1 (underweight), 2 (normal), 3 (overweight)	39	(19.4)	140	(69.7)	22	(10.9)
Diarrhea	DR	1 (YES), 2 (NO)	77	(38.3)	124	(61.7)		
Vomit Vomit and Diarrhea	VOM	1 (YES), 2 (NO) 1 (YES), 2 (NO)	45 37	(22.4) (18.4)	156 164	(77.6) (81.6)		
Change in diet	NCH	1 (YES), 2 (NO)	35	(17.4)	166	(82.6)		
Vaccination	VAC	1 (YES), 2 (NO)	162	(80.6)	39	(19.4)		
Deworming for endoparasites	END	1 (YES), 2 (NO)	146	(72.6)	55	(27.4)		
Deworming for ectoparasites	EXP	1 (YES), 2 (NO)	102	(50.7)	99	(49.3)		
Other animals in the family	OTH	1 (YES), 2 (NO)	99	(49.3)	102	(50.7)		
Symptoms in a member of the family	SM	1 (YES), 2 (NO)	6	(3)	195	(97)		
Contact with stray animals	STR	1 (YES), 2 (NO)	63	(31.3)	138	(68.7)		
Astrovirus	AST	1 (YES), 2 (NO)	29	(15)	172	(85)		
Sapovirus	SAP	1 (YES), 2 (NO)	52	(26)	149	(74)		
Norovirus	NoV	1 (YES), 2 (NO)	0	(0)	201	(100)		

AstV and SaV were detected in 15% and 26% of the examined animals, respectively, while NoV was detected in none of the examined dogs. AstV was detected in 12 dogs with diarrhea and in 18 dogs without diarrhea, while SaV was detected in 19 diarrheic and 33 non-diarrheic dogs, respectively. In 8 dogs, a mixed infection with AstV and SaV was observed.

## Data Availability

Data are contained within the article.

## References

[1] Steiner J.M. (2005). BSAVA Manual of Canine and Feline Gastroenterology.

[2] Gizzi A.B., Oliveira S.T., Leutenegger C.M., Estrada M., Kozemjakin D.A., Stedile R., Marcondes M., Biondo A.W. (2014). Presence of infectious agents and co-infections in diarrheic dogs determined with a real-time polymerase chain reaction-based panel. BMC Vet. Res..

[3] Alves C.D.B.T., Granados O.F.O., Budaszewski R.D.F., Streck A.F., Weber M.N., Cibulski S.P., Pinto L.D., Ikuta N., Canal C.W. (2018). Identification of enteric viruses circulating in a dog population with low vaccine coverage. Braz. J. Microbiol..

[4] Bhatta T.R., Chamings A., Vibin J., Alexandersen S. (2019). Detection and characterisation of canine astrovirus, canine parvovirus and canine papillomavirus in puppies using next generation sequencing. Sci. Rep..

[5] Flynn W.T., Saif L.J. (1988). Serial propagation of porcine enteric calicivirus-like virus in primary porcine kidney cell cultures. J. Clin. Microbiol..

[6] Guo M., Evermann J.F., Saif L.J. (2001). Detection and molecular characterization of cultivable caliciviruses from clinically normal mink and enteric caliciviruses associated with diarrhea in mink. Arch. Virol..

[7] Koci M.D., Schultz-Cherry S. (2002). Avian astroviruses. Avian Pathol..

[8] Martella V., Moschidou P., Buonavoglia C. (2011). Astroviruses in dogs. Vet. Clin. N. Am. Small Anim. Pract..

[9] Stamelou E., Giantsis I.A., Papageorgiou K.V., Petridou E., Davidson I., Polizopοulou Z.S., Papa A., Kritas S.K. (2022). First report of canine Astrovirus and Sapovirus in Greece, hosting both asymptomatic and gastroenteritis symptomatic dogs. Virol. J..

[10] Stamelou E., Giantsis I.A., Papageorgiou K.V., Petridou E., Davidson I., Polizopoulou Z.S., Papa A., Kritas S.K. (2022). Epidemiology of Astrovirus, Norovirus and Sapovirus in Greek pig farms indicates high prevalence of Mamastrovirus suggesting the potential need for systematic surveillance. Porc. Health Manag..

[11] De Benedictis P., Schultz-Cherry S., Burnham A., Cattoli G. (2011). Astrovirus infections in humans and animals: Molecular biology, genetic diversity, and interspecies transmissions. Infect. Genet. Evol..

[12] Rivera R., Nollens H.H., Venn-Watson S., Gulland F.M.D., Wellehan J.F.X. (2010). Characterization of phylogenetically diverse astroviruses of marine mammals. J. Gen. Virol..

[13] Vu D.L., Bosch A., Pintó R., Guix S. (2017). Epidemiology of classic and novel human astrovirus: Gastroenteritis and beyond. Viruses.

[14] Bouzalas I.G., Wüthrich D., Walland J., Drögemüller C., Zurbriggen A., Vandevelde M., Oevermann A., Bruggmann R., Seuberlich T. (2014). Neurotropic astrovirus in cattle with nonsuppurative encephalitis in Europe. J. Clin. Microbiol..

[15] Yi S., Niu J., Wang H., Dong G., Guo Y., Dong H., Wang K., Hu G. (2018). Molecular characterization of feline astrovirus in domestic cats from Northeast China. PLoS ONE.

[16] Arruda B., Arruda P., Hensch M., Chen Q., Zheng Y., Yang C., Gatto I.R.H., Ferreyra F.M., Gauger P., Schwartz K. (2017). Porcine Astrovirus Type 3 in central nervous system of swine with polioencephalomyelitis. Emerg. Infect. Dis..

[17] Blomström A.L., Widén F., Hammer A.S., Belák S., Berg M. (2010). Detection of a novel astrovirus in brain tissue of mink suffering from shaking mink syndrome by use of viral metagenomics. J. Clin. Microbiol..

[18] Bidin M., Bidin Z., Majnaric D., Tisljar M., Lojkic I. (2012). Circulation and phylogenetic relationship of chicken and Turkey-origin astroviruses detected in domestic ducks (Anas platyrhynchos domesticus). Avian Pathol..

[19] Bidin M., Lojkic I., Tisljar M., Bidin Z., Majnaric D. (2012). Astroviruses associated with stunting and pre-hatching mortality in duck and goose embryos. Avian Pathol..

[20] Sajewicz-Krukowska J., Domanska-Blicharz K. (2016). Nearly full-length genome sequence of a novel astrovirus isolated from chickens with ‘white chicks’ condition. Arch. Virol..

[21] Madeley C.R., Cosgrove B.P. (1975). Letter: 28 nm particles in faeces in infantile gastroenteritis. Lancet.

[22] Williams F.P. (1980). Astrovirus-like, coronavirus-like, and parvovirus-like particles detected in the diarrheal stools of beagle pups. Arch. Virol..

[23] Zhu L.A., Zhao W., Yin H., Shan T.L., Zhu C.X., Yang X., Hua X.G., Cui L. (2011). Isolation and characterization of canine astrovirus in China. Arch. Virol..

[24] Martella V., Moschidou P., Lorusso E., Mari V., Camero M., Bellacicco A., Losurdo M., Pinto P., Desario C., Banyai K. (2011). Detection and characterization of canine astroviruses. J. Gen. Virol..

[25] Grellet A., De Battisti C., Feugier A., Pantile M., Marciano S., Grandjean D., Cattoli G. (2012). Prevalence and risk factors of astrovirus infection in puppies from French breeding kennels. Vet. Microbiol..

[26] Castro T.X., Cubel Garcia R.C.N., Costa E.M., Leal R.M., Xavier M.D.P.T., Leite J.P.G. (2013). Molecular characterisation of calicivirus and astrovirus in puppies with enteritis. Vet. Rec..

[27] Choi S., Lim S., Kim Y., Cho Y., Song J., An D. (2014). Phylogenetic analysis of astrovirus and kobuvirus in Korean dogs. J. Vet. Med. Sci..

[28] Martella V., Moschidou P., Catella C., Larocca V., Pinto P., Losurdo M., Corrente M., Lorusso E., Banyai K., Decaro N. (2012). Enteric disease in dogs naturally infected by a novel canine astrovirus. J. Clin. Microbiol..

[29] Wenyan Z., Wang R., Liang J., Zhao N., Li G., Gao Q., Su S. (2020). Epidemiology, genetic diversity and evolution of canine astrovirus. Transbound Emerg. Dis..

[30] Zhou H., Liu L., Li R., Qin Y., Fang Q., Balasubramaniam V.R., Chen Y. (2017). Detection and genetic characterization of canine astroviruses in pet dogs in Guangxi, China. Virol. J..

[31] Caddy S., Goodfellow I. (2015). Complete genome sequence of canine astrovirus with molecular and epidemiological characterization of UK strains. Vet. Microbiol..

[32] Green K.Y., Ando T., Balayan M.S., Berke T., Clarke I.N., Estes M.K., Matson D.O., Nakata S., Neill J.D., Studdert M.J. (2000). Taxonomy of the caliciviruses. J. Infect. Dis..

[33] Schaffer F.L., Soergel M.E., Black J.W. (1985). Characterization of a new calicivirus isolated from feces of a dog. Arch. Virol..

[34] Martella V., Lorusso E., Decaro N., Elia G., Radogna A., D’Abramo M., Desario C., Cavalli A., Corrente M., Camero M. (2008). Detection and molecular characterization of a canine norovirus. Emerg. Infect. Dis..

[35] Villabruna N., Koopmans M.P.G., de Graaf M. (2019). Animals as Reservoir for Human Norovirus. Viruses.

[36] Mesquita J.R., Nascimento M.S. (2012). Molecular epidemiology of canine norovirus in dogs from Portugal, 2007–2011. BMC Vet. Res..

[37] Mesquita J.R., Delgado I., Costantini V., Heenemann K., Vahlenkamp T.W., Vinjé J., Nascimento M.S.J. (2014). Seroprevalence of canine norovirus in 14 European countries. Clin. Vaccine Immunol..

[38] Van Beek J., de Graaf M., Al-Hello H., Allen D.J., Ambert-Balay K., Botteldoorn N., Brytting M., Buesa J., Cabrerizo M., Chan M. (2018). Molecular surveillance of norovirus, 2005–2016: An epidemiological analysis of data collected from the NoroNet network. Lancet Infect. Dis..

[39] Bodnar L., Di Martino B., Di Profio F., Melegari I., Lanave G., Lorusso E., Cavalli A., Elia G., Bányai K., Marsilio F. (2016). Detection and molecular characterization of sapoviruses in dogs. Infect. Genet. Evol..

[40] Li L., Pesavento P.A., Shan T., Leutenegger C.M., Wang C., Delwart E. (2011). Viruses in diarrhoeic dogs include novel kobuviruses and sapoviruses. J. Gen. Virol..

[41] Soma T., Nakagomi O., Nakagomi T., Mochizuki M. (2015). Detection of Norovirus and Sapovirus from diarrheic dogs and cats in Japan. Microbiol. Immunol..

[42] Statista. https://www.statista.com/statistics/515515/dog-population-europe-greece/.

[43] Hieshowcase. https://hieshowcase.com/3939/news/greeces-ever-growing-problem-with-stray-pets/.

[44] Rover. https://www.rover.com/blog/body-condition-score-dog-overweight/.

[45] Jiang X., Huang P.W., Zhong W.M., Farkas T., Cubitt D.W., Matson D.O. (1999). Design and evaluation of a primer pair that detects both Norwalk- and Sapporo-like caliciviruses by RT-PCR. J. Virol. Methods..

[46] Caddy S., Emmott E., El-Attar L., Mitchell J., De Rougemont A., Brownlie J., Goodfellow I. (2013). Serological evidence for multiple strains of canine Norovirus in the UK dog population. PLoS ONE.

[47] Wang Y., Li Y., Cui Y., Jiang S., Liu H., Wang J., Li Y. (2021). Duplex SYBR Green I-based real-time PCR assay for the rapid detection of canine Kobuvirus and Canine Astrovirus. J. Virol. Methods.

[48] Kumar S., Stecher G., Li M., Knyaz C., Tamura K. (2018). MEGA X: Molecular evolutionary genetics analysis across computing platforms. Mol. Biol. Evol..

[49] Greenacre M.J. (1984). Theory and Applications of Correspondence Analysis.

[50] Tenenhaus M., Young F.W. (1985). An analysis and synthesis of multiple correspondence analysis, optimal scaling, dual scaling, homogeneity analysis and other methods for quantifying categorical multivariate data. Psychometrika.

[51] Moschidis S., Markos A., Thanopoulos A.C. (2022). “Automatic” interpretation of multiple correspondence analysis (MCA) results for nonexpert users, using R programming. Appl. Comput. Inform..

[52] Reddy K.C., Vinzamuri B. (2018). A Survey of Partitional and Hierarchical Clustering Algorithms.

[53] (2019). The R Project for Statistical Computing. https://www.r-project.org.

[54] Lê S., Josse J., Husson F. (2008). FactoMineR: An R Package for Multivariate Analysis. J. Stat. Soft..

[55] https://www.python.org/.

[56] www.scikit-learn.org/stable/.

[57] Davidson I., Stamelou E., Giantsis I.A., Papageorgiou K.V., Petridou E., Kritas S.K. (2022). The Complexity of Swine Caliciviruses. A Mini Review on Genomic Diversity, Infection Diagnostics, World Prevalence and Pathogenicity. Pathogens.

[58] Müllner D. (2011). Modern hierarchical, agglomerative clustering algorithms. arXiv.

[59] Florensa D., Mateo-Fornés J., Solsona F., Pedrol A.T., Mesas Julió M., Piñol R., Godoy P. (2022). Use of Multiple Correspondence Analysis and K-means to Explore Associations Between Risk Factors and Likelihood of Colorectal Cancer: Cross-sectional Study. J. Med. Internet Res..

[60] Costa P.S., Santos N.C., Cunha P., Cotter J., Sousa N. (2013). The Use of Multiple Correspondence Analysis to Explore Associations between Categories of Qualitative Variables in Healthy Ageing. J. Aging Res..

[61] Moreno P.S., Wagner J., Mansfield C.S., Stevens M., Gilkerson J.R., Kirkwood C.D. (2017). Characterisation of the canine faecal virome in healthy dogs and dogs with acute diarrhoea using shotgun metagenomics. PLoS ONE.

[62] Strain E., Kelley L.A., Schultz-Cherry S., Muse S.V., Koci M.D. (2008). Genomic analysis of closely related astroviruses. J. Virol..

[63] Donato C., Vijaykrishna D. (2017). The Broad Host Range and Genetic Diversity of Mammalian and Avian Astroviruses. Viruses.

[64] Wolfaardt M., Kiulia N.M., Mwenda J.M., Taylor M.B. (2011). Evidence of a recombinant wild-type human astrovirus strain from a Kenyan child with gastroenteritis. J. Clin. Microbiol..

[65] Walter J.E., Briggs J., Guerrero M.L., Matson D.O., Pickering L.K., Ruiz-Palacios G., Berke T., Mitchell D.K. (2001). Molecular characterization of a novel recombinant strain of human astrovirus associated with gastroenteritis in children. Arch. Virol..

[66] Ulloa J.C., Gutiérrez M.F. (2010). Genomic analysis of two ORF2 segments of new porcine astrovirus isolates and their close relationship with human astroviruses. Can. J. Microbiol..

[67] Chae S.B., Jeong C.G., Park J.S., Na E.J., Oem J.K. (2023). Detection and Genetic Characterization of Astroviruses in Brain Tissues of Wild Raccoon Dogs. Viruses.

[68] Roach S.N., Langlois R.A. (2021). Intra- and Cross-Species Transmission of Astroviruses. Viruses.

[69] Hata A., Kitajima M., Haramoto E., Lee S., Ihara M., Gerba C.P., Tanaka H. (2018). Next-generation amplicon sequencing identifies genetically diverse human astroviruses, including recombinant strains, in environmental waters. Sci. Rep..

